# A new framework for mental illnesses diagnosis using wearable devices aided by improved convolutional neural network

**DOI:** 10.1038/s41598-025-07245-6

**Published:** 2025-07-23

**Authors:** Hend S. Saad, John F. W. Zaki, Mohamed M. Abdelsalam

**Affiliations:** 1 Computers and Control Systems Engineering Department Faculty of Engineering, Mansoura University, Mansoura, 35516 Egypt; 2 Raya Higher Institute of Management and Foreign Trade, New Damietta, Damietta Egypt; 3 Faculty of Engineering, Mansoura National University, Gamasa, Mansoura Egypt

**Keywords:** Mental health disorders, Markov transition field, Convolutional neural network, Schizophrenia, Depression, Computational biology and bioinformatics, Data processing, Machine learning

## Abstract

Stress inherent in the modern world is considered one of the main causes of Mental Health Disorders (MHDs) that spread in every country around the world. These mental and behavioral problems primarily affect the mind and brain that change emotions and perception, especially if they are not diagnosed or treated early. MHD diseases are difficult to be distinguished from each other because they come in many forms with different severity of symptoms and different periods of suffering. A person’s bioactivity can be measured by the wearable technology such as smart watches that become more advanced and widely spread. A new framework based on analyzing the motor activity data measured by smart watches is presented to diagnose mental illnesses such as schizophrenia and depression as well as analyze complex behavioral patterns. The framework encodes the behavioral time series data into image patterns using modified Markov Transition Field. These images have been processed using a modified Convolutional Neural Network and an attention pooling approach for the classification of depression and schizophrenia patients. The proposed system achieved an accuracy of 96.6% in schizophrenia and 94.85% in depression as a precision of 92.1% in schizophrenia and 96.77% in depression. The proposed system is able to analyze the motor activity of individual and able to diagnose the mental illness. The results of the proposed system are superior to those used in detecting mental health disorders.

## Introduction

Mental Health Disorders (MHDs) are increasing globally due to the increasing stresses of modern life and incidence rates are increasing further in developing and developed countries^1,2^. The MHD diseases come in a variety of types, each with a unique severity of symptoms and duration of suffering. These diseases are difficult to detect and can be confused with each other. There can be difficult repercussions if these disorders are underestimated or handled incorrectly. The MHDs have a direct impact on the physical and emotional well-being of their patients, as well as those close to them and the general public^3,4^.

Depression is a common mental illness that impairs mental health and leads to a loss of the desire to live. Somatic, volitional, cognitive, and affective are the different symptoms of this disease^5–7^. To be classified as depressed, patients must experience these symptoms for at least two weeks. Moreover, since depression is closely related to motor activity and can impair an individual’s ability to perform daily household chores, it is one of the main causes of occupational impairment^8,9^.

The International Statistical Classification of Diseases and Related Health Problems (ICD-10) and The Diagnostic and Statistical Manual of Mental Disorders (DSM-5) classifications reveal that bipolar depression and unipolar depression lack distinct symptomatic criteria for consistent differentiation^10^. Therefore, the best time to diagnose Major Depression Disorder (MDD) is during a depressive episode, which can occur in both bipolar and unipolar patients. On the other hand, manic depression, a term used to describe bipolar disorder, occurs when mania and depression alternate^11,12^. Another mental illness that causes severe mood changes in a person is bipolar depression. For the majority of patients with bipolar disorder, the origin of manic or depressive episodes is unclear^13^.

Schizophrenia is another mental disorder that characterized by delusions or hallucinations in patients. It can be classified as positive, accompanied by aggressive behavior, or negative, with depressive symptoms^14,15^. Patients with schizophrenia have changes in their verbal, processing speed, functional, memory and due to changes in the prefrontal and parietal-temporal cortex^16^. Both depression and schizophrenia are severe MHDs characterized by severe symptoms. Several researches have revealed that these two disorders share symptoms, making it challenging for medical professionals to diagnose the condition and administer the appropriate course of therapy. Thus, it is crucial and necessary to discover MHDs early, rather than only relying on symptoms, in order to prevent or lessen negative consequences for patients in the future^17,18^.

Patients with MHDs need continuous monitoring by a medical professional over a long period of time, so behavioral patterns of daily activity present challenges as a clinical diagnostic tool^19^. This problem can be solved by the latest developments in Internet of Things (IoT) sensors, especially wearable technologies^9^. Providing objective measurements, low cost, and patient convenience are the major advantages of this approach. Actigraph IoT devices can be used to record a continuous and one-dimensional signal along with the wearer’s activities, based on their built-in accelerometers^20^. They are very similar to watches and often combined with watch functionality. In the mental health field, data from these sensors have been used to support the diagnosis of Attention-Deficit/Hyperactivity Disorder (ADHD)^21^, depression^22^, and schizophrenia^23^.

One of the main challenges that faces this study is the small size of the used dataset (depresjon and psykose). So, a segmentation process has been used by utilizing a variable time window to capture the time series dataset to obtain different motor activity patterns. After segmentation, each segment has been transformed into an image using Markov Transition Field (MTF). The goal of MTF is to convert activity sequences into image patterns that reflect transitions between states, helping the model to capture more detailed information. After conversion, resize all images to ensure that all images have the same dimensions, which is essential for the model to process them correctly. Then, applying a normalization procedure to all the images to scale values between 0 and 1, which makes the model learn faster and more accurately by stabilizing the training. Finally, Modified Convolutional Neural Networks (CNN) have been used for the process of classification with more Conv2D layers and Dropout layers to reduce the overfitting.

The structure of the research consists of the following: Section “Motivation and contributions” describes the motivation and contribution of the research. Section “Related works” includes a comprehensive literature review, which summarizes previous research and basic algorithms. The materials and dataset used are described in Section “Materials”. The proposed method, which emphasizes the unique algorithm, is introduced in Section “Methods”. The implementation results are discussed in Section “Results and discussions”, which demonstrates the success of the proposed strategy. The work is concluded, and further research topics are proposed in Section “Results and discussions”.

## Motivation and contributions

### Motivation

1. In contrast to real-world clinical practice, machine learning-based diagnostic tools for identifying patients with established psychotic disorders have limited clinical utility because early detection of the disease may be unexpected and treatment decisions are sometimes pending.
2. The dataset based on motor activity for classifying depression and schizophrenia disorders is very low.
3. Most of the research used the dataset as time series only.
4. No sufficient high classification accuracy for depression and schizophrenia disorders.

### Contributions

The three main contributions of this study are as follows:

1. Classify the mental health disorders as depression and schizophrenia using wearable devices based on Markov Transition Field method.
2. The used dataset contains only 55 participants for depression (depresjon) and 54 participants for schizophrenia (psykose). Therefore, Split the dataset into different time window (TW) sizes to capture the motor activity changes.
3. Achieve superior classification performance compared to current state-of-the-art methods when tested under the same experimental conditions.

## Related works

Numerous studies have already been done in the field of Mental Health Monitoring Systems (MHMS). Tables 1 and 2 show some previous studies that used Machine Learning (ML) to analyze depression and schizophrenia disorders, respectively.

Table 1 shows several research that discussed to detect depression disorder. In addition to publish the so-called “depresjon” dataset, The authors in^24^ used various ML techniques and contrasted the outcomes. With an accuracy of 72.7%, linear Support Vector Machine (SVM) was shown to be the most effective technique. The so-called zeroR-classifier (assignment of majority class) had a 58% accuracy rate. The authors stressed the importance of advanced feature engineering. A specialized end-to-end model was developed in^25^ to diagnose depression from audio datasets, achieving an accuracy of 83%. In^27^, a method for automatic depression diagnosis using deep convolutional neural networks was proposed. This approach hierarchically learned discriminative features by processing Mel-Frequency Cepstral Coefficients (MFCC) feature maps extracted from preprocessed speech and training them using residual CNNs. To optimize recognition accuracy, the model adjusted the network’s layers and nodes accordingly. It can be difficult to diagnose mental health illnesses automatically using voice recognition while maintaining the patient’s identity. In a separate study, researchers in^28^ introduced an adversarial disentanglement technique to separate speaker identity from depressive traits. The model was designed to classify depression while ensuring it remained unaffected by speaker identity by maximizing speaker prediction loss and minimizing depression prediction loss during training.

The “Normalized Statistical Features” nighttime dataset in^29^ demonstrated the predictive performance of machine learning models for the Control and Condition groups. The Random Forest Classifier achieved an 83.41% accuracy rate which surpassed the performance of the four other refined models followed by the Gradient Boosting Classifier which achieved 82.03%. A model was utilized in^30^ to analyze patient representative data and determine the presence of depression. By examining speech characteristics of individuals with depressive illness using the DAIC-WOZ dataset, researchers conducted an in-depth study on speech-assisted depression diagnosis, achieving an accuracy of 87%. The development of a new Machine Speech Chain Model for Depression Recognition (MSCDR) aimed to increase recognition accuracy^31^. The model successfully detected depressed speech patterns regardless of who spoke to them and replicated the natural communication flow beginning with speech production and ending with perception. The MSCDR executed speech perception analysis through Mel-Frequency Cepstral Coefficients (MFCC) and performed speech synthesis using Linear Predictive Coding (LPC). Following this, a one-dimensional convolutional neural network (CNN) combined with a long short-term memory (LSTM) network was employed to capture both intra-segment and inter-segment dynamic depression features for classification. To achieve 90% accuracy in automatically detecting depression from audio signals, a novel approach integrating deep learning with traditional audio features was explored in^33^. The researcher in^34^ investigated the application of wearable biophysiological data to identify depression. The multimodal wristbands, which recorded heart rate, skin conductance, and acceleration, provided data from 58 depressed individuals and 58 non-depressed controls. The authors used ML algorithms such as Random Forest to process the static and dynamic aspects of the data and obtained a classification success of 90% given 6-hour data segments. The findings show the promise of this approach as a monitoring technology for the early detection and monitoring of depression in daily life, providing new opportunities for mental health care using wearable technologies.

**Table 1:** Comparison of different techniques for diagnosing depression from previous works.

Ref.	ML technique	Dataset	Accuracy	Cons
^24^	Several ML techniques like: Linear SVM, Naive Bayes, Nearest Neighbors, Decision Tree, Neural Net, ZeroR baseline, AdaBoost.	Depresjon	The best method is Linear SVM with accuracy: 72.7%	There is need for sophisticated feature engineering.
^25^	CNN	DAIC-WOZ^26^	Accuracy: 83%	Accuracy is low and the used ML technique is old
^27^	CNN (ResNet-34)	Northwest Normal University	Accuracy: 77%	
^28^	DepAudioNet	DAIC-WOZ	Accuracy: 74%	Noise in the dataset
^29^	Random Forest (RF), Gradient Boosting, K-Neighbors, SVM	Depresjon	The highest accuracy: 83.41%	Exploring additional feature extraction techniques is required
^30^	CNN	DAIC-WOZ	Accuracy: 87%	Low accuracy as well as the outdated of the used method.
^31^	CNN (CNN-LSTM)	DAIC-WOZ and MODMA^32^	Accuracy: 77% for DAIC-WOZ And 86% for MODMA	The small sample size may restrict its applicability in clinical practice to some extent.
^33^	A novel CNN network was used to extract both Mel-Frequency Cepstral Coefficients (MFCC) and Spectrogram features from an audio file.	MODMA	Accuracy: 90%	Accuracy is still low
^34^	RF, SVM, K-NN, Linear Discriminant Analysis (LDA)	DAPPER dataset^35^	Accuracy: (90.0 ± 1.7%)	The accuracy is relatively low, which may limit its reliability in practical applications.

Table 2 also shows several research that discussed to detect schizophrenia disorder. Deep learning is a very strong technique for extracting, integrating, and utilizing information from multimodal data (such as simultaneous Electroencephalogram (EEG)/ Functional Magnetic Resonance Imaging (fMRI)) to detect schizophrenia illness. A small 1D convolutional Artificial Neural Network (ANN) was employed in^36^ on brain MRI/fMRI maps and were able to achieve accuracy levels that were on par with conventional machine learning techniques (84% accuracy). 3D convolutional ANN was used in^37^ using structural brain MRI and it performed satisfactorily in terms of classification in a different set of patients at a lower stage of the illness (88.6% accuracy when pooling sites; 70% accuracy when evaluating on held-out site). Motor activity data enables the examination of complex behavioral patterns, including the diagnosis of mental illnesses. The motor activity-based Psykose dataset was used in^39^ using Logistic Regression (LR), SVM, and RF methods to diagnose schizophrenia. The lowest accuracy is 0.80 ± 0.06 for SVM and the highest is 0.89 ± 0.07 for RF. To overcome the limitations of feature extraction-based methods, Khari et al.^40^ proposed an automated schizophrenia identification method that combines CNN and time-frequency analysis. EEG signals were processed using three different techniques: Smooth Pseudo Wigner-Ville Distribution (SPWVD), short-term Fourier transform, and continuous wavelet transform. These methods generated SPWVD-based time-frequency representation (TFR) plots, providing a detailed visualization of signal variations over time. The accuracy of the method, as determined by the CNN model and SPWVD-based TFR, was 93.36%. The method’s dependence on the choice of experimental parameters and additional memory requirements were some of its drawbacks.

In order to examine inpatient violence among criminal patients with schizophrenia spectrum disorders (SSDs), researchers in^42^ applied a machine learning algorithm to a dataset of 370 individuals. Support Vector Machine (SVM) performed better than the other machine learning techniques examined, with an AUC of 0.87 and a balanced accuracy of 77.6%. The authors of^43^ suggested a simple 3D CNN-based technique for diagnosing schizophrenia (SZ) from MRI data. Following the extraction of MRI scan information, the model was categorized using an ensemble learning bagging classifier. Three benchmark databases—fBRINPhaseII, COBRE, and MCICShare—were used to assess the method. The model achieved the highest accuracy of 92.22% and sensitivity of 94.44%, in contrast to existing methods. The higher computational cost of training 3D models was one of the shortcomings of the study. The binary labels present in the MRI data were used to train the Deep Learning (DL) algorithm, which simplifies the fact that people can have multiple diseases at the same time and that many mental health problems develop along with them. In addition, the model was prone to changing its hyperparameters. A novel deep learning approach was proposed in^44^ for intelligent detection of SZ and ADHD in Resting-State Functional Magnetic Resonance Imaging (rs-fMRI) using the University of California Los Angeles dataset^45^. Grey Wolf Optimization (GWO), particle swarm optimization, and genetic algorithms were used to further enhance Interval Type-2 Fuzzy Regression (IT2FR) during the classification step. According to experimental results, the IT2FR technique had the maximum accuracy of 72.71% when optimized using GWO.

Furthermore, the study in^46^ sought to improve diagnostic techniques’ sensitivity and specificity in order to identify and treat people at risk for pathological schizotypal disease as soon as possible. For categorization, a number of machine learning models were employed, such as Light Gradient Boosting (LGB), Random Forest (RF), and Logistic Regression (LR). The findings showed that LR outperformed both with an accuracy of 83%, RF reached 75%, and LGB reached 79%.

**Table 2:** Comparison of different techniques for diagnosing schizophrenia from previous works.

Ref.	ML technique	Dataset	Accuracy	Cons
^36^	1D convolutional ANN applied to the concatenated brain maps.	Magnetic resonance images (MRI)	84%	The accuracy is low, and the method used is outdated.
^37^	3D convolutional ANN	873 structural MRI datasets^38^	88.6% accuracy when pooling sites; 70% accuracy when evaluating on held out site.	1- Each MRI scan used to train the deep learning algorithm was assigned a binary label, categorizing it as either normal or schizophrenia. 2- The datasets lacked detailed information regarding the specific aspects of the illness.
^39^	Support Vector Machine (SVM), Random Forest (RF), and Logistic regression (LR).	Psykose	The lowest accuracy is 0.80 ± 0.06 for SVM and the highest is 0.89 ± 0.07 for RF	The methods used here are relatively simple.
^40^	CNN	EEG data^41^	93.36%	The reliance on empirical selection of parameters and additional memory requirements
^42^	Support Vector Machine (SVM)	Unpublic dataset of 370 patients	77.6%	The method used is relatively simple.
^43^	A lightweight 3D convolutional neural network (CNN)	MRI images	92.22%	The increased computational cost is associated with training 3D models.
^44^	Interval type-2 fuzzy regression (IT2FR)	The University of California Los Angeles dataset^45^	72.71%	Accuracy is still low
^46^	LR, RF, Light Gradient Boosting (LGB)	Measurement data, collected by Maczák et al.^47^	LR (79%), LGB (83%), and RF (75%).	The small sample size increases the likelihood of random effects.

## Materials

### Specification of the used device for running the code

*Hardware*:

Processor: Intel(R) Core(TM) i5-4200 M CPU @ 2.50 GHz 2.50 GHz.

Installed memory (RAM): 16.0 GB.

*Software*:

Windows: 10 Pro.

System type: 64-bit Operating System, x64-based processor.

### Data description

The two datasets used in this study, Depresjon^24^ and Psykose^48^, comprise motor activity data collected using Actiwatch wristwatches (model AW4) developed by Cambridge Neurotechnology Ltd. in England. This device features a specialized piezoelectric accelerometer that measures movement intensity, duration, and magnitude across the x, y, and z axes. It captures motion data at a frequency of 32 Hz, producing an integer value every minute that corresponds to the movement’s intensity.

#### Depresjon dataset

The dataset consists of motor activity measurement data from 23 patients with unipolar and bipolar depression (condition group). During the data collection period, 18 participants were in outpatient clinics and 5 were in the hospital. At the beginning and end of the motor activity recordings, the therapist used the Montgomery-Asberg Depression Rating Scale^24^ to score the severity of ongoing depression. The dataset al.so includes motor activity measurement data from 32 non-depressed participants (control group), which included 4 former patients with no current psychiatric symptoms, 5 students, and 23 hospital staff. The time longitude for each participant was varying, with an average of 12.6 days.

#### Psykose dataset

The dataset consists of Actigraph data from 22 psychotic patients admitted to a long-term open psychiatric ward at Haukeland University Hospital. All participants had been diagnosed with schizophrenia and were undergoing antipsychotic treatment. The group comprised 19 males and 3 females, with an average age of 46.2 ± 10.9 years (ranging from 27 to 69 years). The mean age at the onset of hospitalization was 24.8 ± 9.3 years, with a range of 10 to 52 years. Clinical practitioners diagnosed the patients using a semi-structured interview based on DSM-IV criteria^48^. Among them, 17 individuals were identified with paranoid schizophrenia, while the remaining five did not exhibit any specific schizophrenia subtype and showed no signs of paranoia.

The dataset also includes motor activity assessment data from 32 healthy individuals, consisting of 23 hospital staff members, 5 nursing students, and 4 individuals selected from a general practitioner’s clinic. None of the participants had a history of psychotic or emotional disorders. The group had an average age of 38.2 ± 13.0 years, ranging from 21 to 66 years, and comprised 12 males and 20 females. While the gender distribution was not identical between groups, previous research on motor activity in mental health has found no significant gender-based differences in activation. On average, participants in both the case and control groups used motor activity measurement devices for 12.7 days.

The dataset used in this study has been collected from motor activity data from patients with depression and schizophrenia, so it is important to consider concept drift and model drift over time. Indeed, patients’ behavior and patterns may change in response to treatment, relapses, or external environmental influences, causing the relationship between motor activity and mental health status to change over time. Model drift may also result from the evolution of the underlying distribution (of the data) due to device updates, sensor wear, or even seasonal/daily variation in activity.

So, validating model performance has been proposed on a validation set as a function of training, examining whether there are sudden and anomalous drops in prediction reliability or other quality metrics. Furthermore, statistical change detection methods, such as KL divergence, the population stability index (PSI), and moving window analysis, can also be used to detect changes in the data distribution.

## Methods

**Fig. 1:**
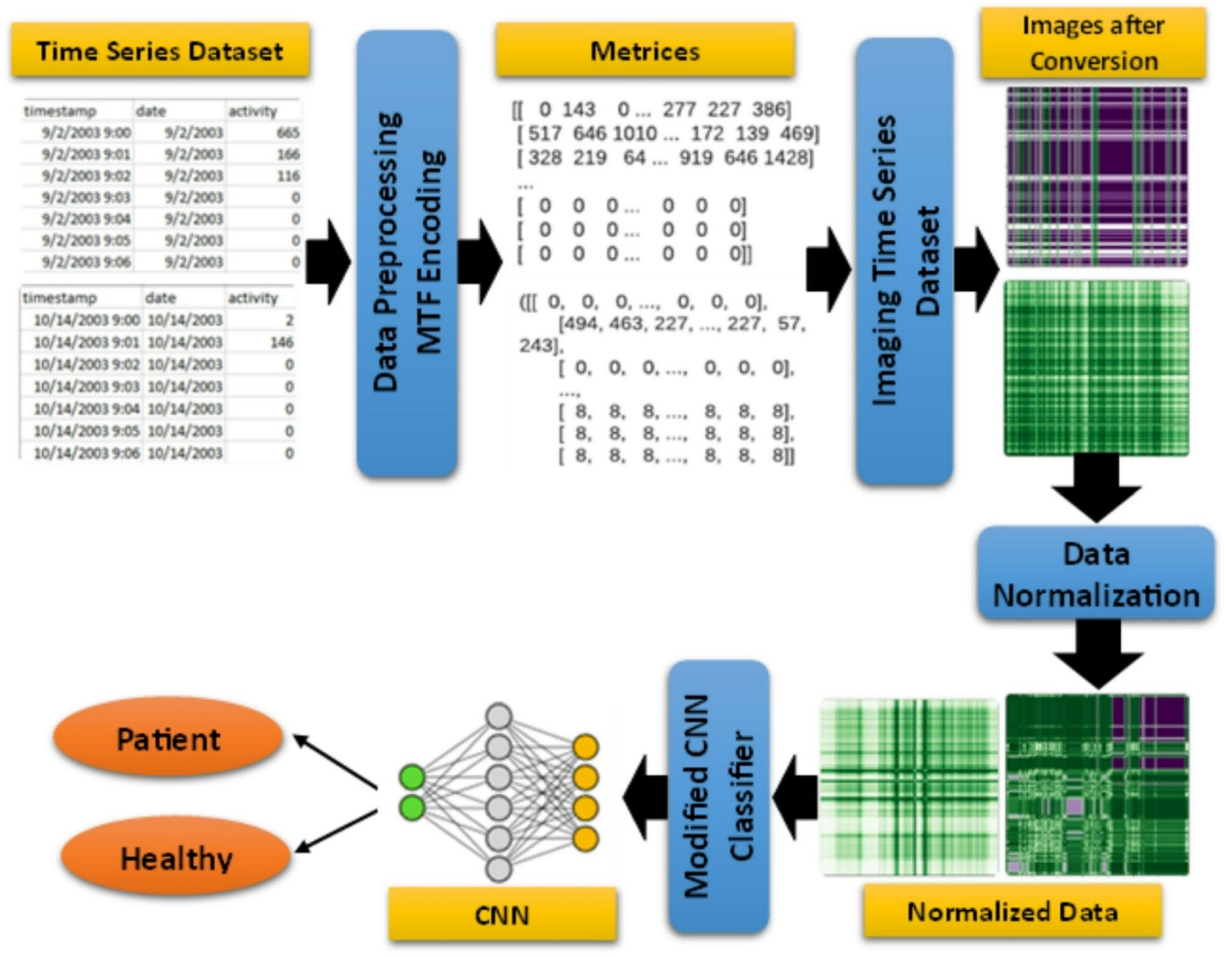
The workflow of the proposed framework.

The workflow of the proposed method is illustrated in Fig. 1. The first stage identifies the source from which the motor activity records of patients or participants are collected. Data preprocessing provides a detailed explanation of how a new dataset is created from the original data by using MTF for imaging time series data. This stage also entails employing a method to correct class imbalance in the data in order to carry out future normalization. Next, the evolution of the 2D-CNN architecture is described, including important elements like the activation function, number of epochs, batch size, feature extraction filter size, pooling techniques, and convolutional layer count. The suggested convolutional neural network and a deep neural network with different depth levels are then used to assess the differences between the two networks using an analytical classification approach. Several classification models are assessed using performance metrics, such as accuracy (ACC), precision, recall, and F1-score, in order to finish the validation process.

### Data preprocessing

#### Imaging time series

In this section, the encode transforms the input motor activity dataset (depresjon and psykose) into images using Markov Transition Field (MTF).

##### Markov transition field (MTF)

The Markov Transition Field (MTF) method is based on the idea of mapping time series into network-like structures, as done in^49^. MTF takes the idea of Markov chains and transforms sequential data into two-dimensional representations that can preserve important temporal structures. The MTF technique extends the standard Markov Transition Matrix by taking into account time dependence and modeling those relationships in a discretized way; this approach provides a more elaborate, high-dimensional feature space. To improve the overall quality of this transformation and reduce computational load, we use fuzzy kernel pooling. Fuzzy kernel pooling improves the MTF matrix by reducing the weight of transition probabilities within local patches and calculating a weighted average, typically using a uniform or Gaussian kernel. This process reduces noise and gives us a clearer picture of how things change over time. This denoising is accomplished by focusing on the most statistically significant transition patterns within a shorter time frame. Ultimately, the MTF transform produces a structured 2D image that preserves both the frequency and sequence of changes seen in the original time series data. Images produced by MTF are well-suited to deep learning models, particularly convolutional neural networks (CNNs), where hierarchical spatial feature extraction thrives. MTF’s ability to structure sequential data into an image with meaningful temporal features allows subsequent tasks, such as emotion recognition, physiological signal analysis, and mental health monitoring, to be more accurately and quickly classified or analyzed^50^.

For a given time, series X quantile bins *Q* are created, and each x_i_​ is assigned to its corresponding bin q_j_ (where j belongs to the range [1, Q]). Similar to a first-order Markov chain, transitions between quantile bins over time are counted to generate a weighted adjacency matrix W of size (Q × Q). The element w_i, j_ represents the frequency with which a point in quantile q_j_ is followed by a point in quantile q_i_. After normalization using $\:{\sum\:}_{j}{w}_{ij}=1$ the Markov transition matrix W is obtained. This matrix remains independent of the distribution of X and the temporal dependencies of time steps t_i_. However, eliminating time dependency results in a substantial loss of information within W, as demonstrated by our experimental findings. To address this issue, the Markov transition field (M) is defined as follows^51,52^:

1 $$\:M=\begin{array}{ccc}{w}_{\left.ij\right|{x}_{1} \in {q}_{i},\:{x}_{1}\in{q}_{j}}&\:\cdots\:&\:{w}_{\left.ij\right|{x}_{1}\in{q}_{i},\:{x}_{n}\in{q}_{j}}\\\:{w}_{\left.ij\right|{x}_{2}\in{q}_{i},\:{x}_{1}\in{q}_{j}}&\:\cdots\:&\:{w}_{\left.ij\right|{x}_{2}\in{q}_{i},\:{x}_{n}\in{q}_{j}}\\\:\begin{array}{c}⋮\\\:{w}_{\left.ij\right|{x}_{n}\in{q}_{i},\:{x}_{1}\in{q}_{j}}\end{array}&\:\begin{array}{c}\ddots\:\\\:\cdots\:\end{array}&\:\begin{array}{c}⋮\\\:{w}_{\left.ij\right|{x}_{n}\in{q}_{i},\:{x}_{n}\in{q}_{j}}\end{array}\end{array}$$

To construct a Q × Q Markov transition matrix (W), the data (magnitude) is segmented into Q quantile bins. The temporal axis is represented by time stamps $\:i$ and $\:j$, corresponding to quantile bins q_i_ and q_j_ (q € [1,Q]). In the Markov Transition Field (MTF), M_ij_ denotes the transition probability from q_i_ to q_j_. This approach ensures that temporal positions are taken into account, allowing the MTF matrix to be propagated into matrix W, which retains the transition probabilities along the magnitude axis.

**Fig. 2:**
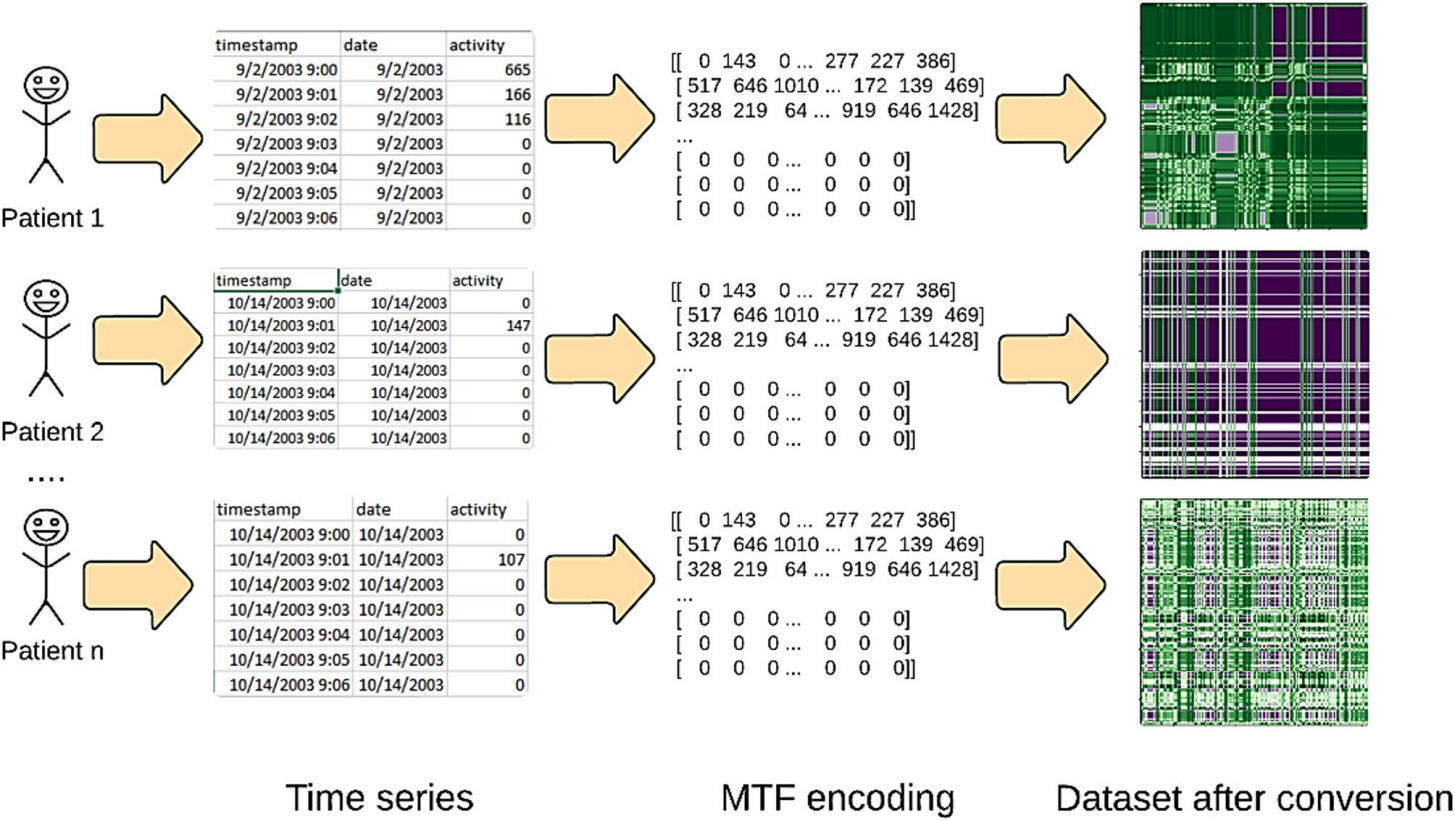
Imaging time series for depresjon dataset using MTF encoding.

**Fig. 3:**
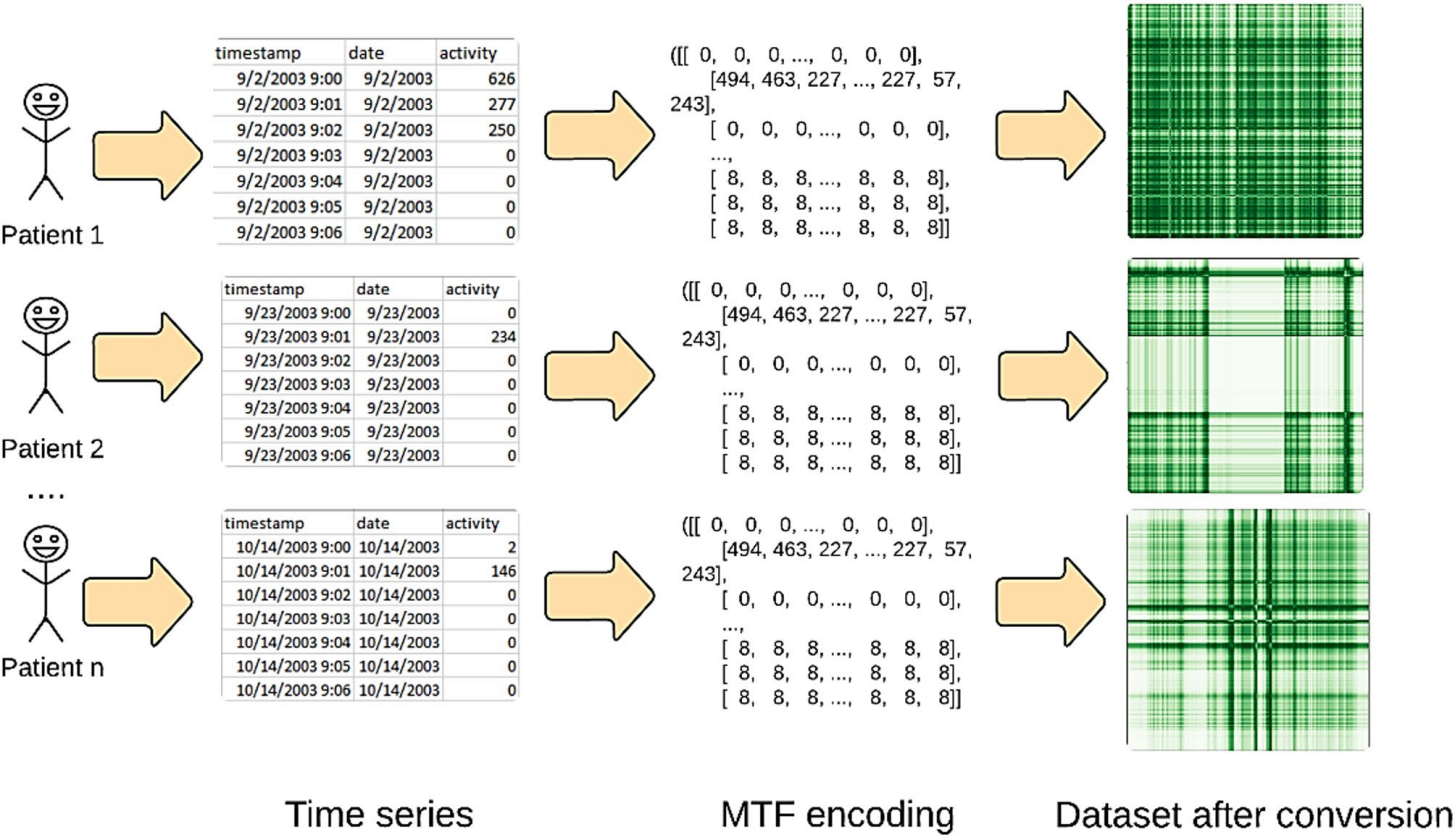
Imaging time series for psykose dataset using MTF encoding.

The MTF M actually encodes the multi-span transition probabilities of the time series by allocating the probability at each pixel M_ij_ from the quantile at time step $\:i$ to the quantile at time step $\:j$. The transition probability between the positions with time interval k is indicated by the notation $\:{M}_{\left.i,j\right|\left|i-j\right|=k}$. For example, $\:{M}_{\left.i,j\right|j-i=1}$ uses a skip step to show how the transition process moves along the time axis. The main diagonal M_ii_ is a specific instance where the probability at each time step $\:i$ from each quantile to itself (the self-transition probability) is captured by k. By averaging the pixels in each non-overlapping $\:m\times\:m$ patch with the blurring kernel $\:{\left\{\frac{1}{{m}^{2}}\right\}}_{m\times\:m}$, the size of the MTF is reduced and the computational efficiency is improved. In other words, the transition probabilities in each subsequence of length $\:m$ are integrated^53,54^. The procedure for encoding the time series to MTF for depresjon and psykose datasets is shown in Figs. 2 and 3, respectively.

### Classification analysis

The extensive use of deep learning for classification across a range of application scenarios has led researchers to propose novel structures based on neural networks^55,56^. This assumption has led to the concept of presenting a healthcare prediction system that incorporates coding methods using neural networks.

#### Convolution neural network

Convolutional neural networks (CNNs) are among the most important algorithms used in deep learning for any analysis involving visual data, due to their high efficiency and outstanding performance. When discussing convolutional neural networks, it is clear that CNNs are more effective due to their parameters, such as joint weights and local receptive fields. These features improve performance while reducing the computational cost of the model^57^. These facts explain the dominance of CNNs in the image recognition and object tracking markets. In recent years, models based on convolutional neural networks (CNNs) have achieved remarkable performance in multidisciplinary projects, in addition to analytical chemistry, for example, such as pattern recognition and predictive analytics^58^.

The core architecture of CNN consists of convolutional layers, activation functions, pooling, and fully connected layers. All convolutional layers contain multiple learnable filters (kernels), which then slide over the input data to focus on important spatial features. Initially, features in the input are detected as simple features such as edges and corners. As the network progresses, these layers begin to focus on more complex and abstract things relevant to the task. An activation layer is a function that introduces nonlinearity to the network often using functions like Rectified Linear Unit (ReLU). This is necessary to enable the model to learn from a wide range of input patterns. A pooling layer (such as max pooling) is used to downsample the feature maps, thereby reducing their dimensionality and computational burden while preserving important spatial features. Finally, the extracted feature sets are passed through one or more fully connected layers to produce the final output, which can be any of the following: classification score, prediction score, or regression score, depending on the application^59^.

In particular, the proposed model is built on 2 classes (Patient and Healthy) of health state predictions. In order to find temporal correlations within different time periods, patient attributes are first evaluated, which indicate the health status of the person. Each attribute is then represented as a time series in Matrix using MTF coding.

#### Creating the CNN model

In this paper, two convolutional neural network (CNN) models for image classification tasks are designed and implemented. The first model has simple architecture, whereas the second model has more layers to improve efficiency and generalization. Details about the architecture of each model, the functions of various layers, and the optimization strategies used to get successful learning results are explained.

#### The model architecture

##### First model: simple CNN

The first model of CNN consists of a stack of Conv2D and MaxPooling2D layers. Multiple filters are applied by the Conv2D layers to home in on certain features of the input image. Take for instance the image is convolved over with a filter size of (5, 5), it allows the model to more readily recognize features like edges and textures. 32 filters are used in this layer, as shown by the setting ‘filters = 32’. This does assist in capturing complex details, but it also increases the demand for computing power. Methodology used to achieve this is Padding=’same’ which guarantees a consistent output with the input in respect to spatial dimensions.

The Conv2D layers utilize the Rectified Linear Unit (ReLU) activation function, which assigns zero to negative values to break symmetry and introduce non-linearity into the model. As a result, this activation function improves the efficiency of the training process.

- **MaxPooling Layer**: Subsequent to the application of Conv2D layers, the MaxPooling2D layers are added in order to reduce the spatial dimensions of the feature maps. These layers achieve this by performing a max pooling operation over the feature maps, for example with window size (2, 2), allowing for the size of the feature maps to be reduced by a factor of two. The tradeoff is that reduction will reduce the number of computations required while preserving the most critical components of the data which is true.
- **Flatten and Dense Layers**: The convolutional layer outputs are multi-dimensional arrays which require conversion to a one-dimensional vector via a Flatten layer. This change renders the data compatible with fully connected (Dense) layers which are for example used to implement a Dense layer ‘packed’ with units = 256 scalar such that the ‘higher’ neuron is able to fit onto higher dimensional neurons and all the patterns and relations extracted from this layer. ReLU activation is also applied to the intermediate Dense layers. The last Dense layer has an output of probabilities in the range of 0 to 1, enabling it to be used in binary classification problems as it has been fitted with a sigmoid activation function.

##### Second model: deeper CNN

This architecture has more depth as it has more Conv2D layers and more Dropout layers to prevent overfitting and improve generalization. The extra Conv2D layers improve feature extraction. In order to enhance the model capabilities, Dropout was used in which 50% of neurons are turned off at random to lessen overfitting each training session.

**Fig. 4:**
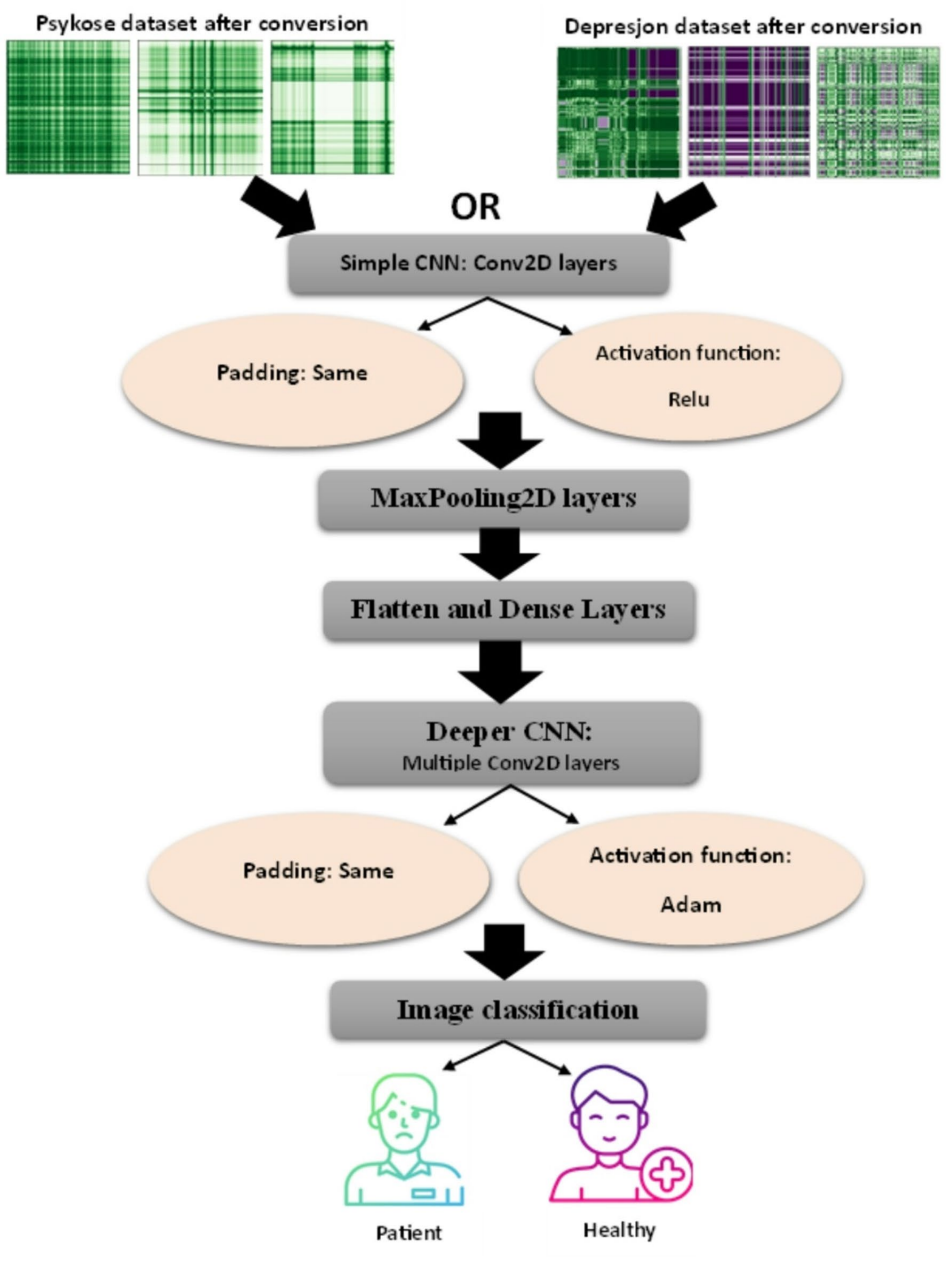
Illustration of the proposed CNN architecture.

- **The Optimization techniques**: Adam optimizer was selected as the optimization algorithm because of its self-adjusting learning rates which incorporate advantages of both SGD and RMSprop. Binary cross entropy loss function is utilized in measuring the error of the predictions and accuracy is recorded as the measure of performance that is being optimized all through the training. With such a setup, the models are able to train and generalize the image features well, making them suitable for good classification performance. Figure 4 presents the proposed architecture of CNN.

### Validation

The Depresjon and Psykose datasets are used to assess prediction performance. For every patient, actigraphic data gathered over time is shown. In terms of pre-processing, following an initial feature selection phase, the most influential attributes were chosen. All attribute’s values were scaled between 0 and 1. The features that are not important for the classification task are removed through the first step, feature selection.

#### Evaluation metrics

**Fig. 5:**
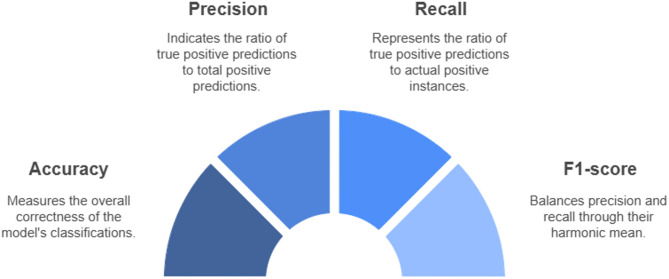
Chart of evaluation metrics.

The performance evaluation is conducted using metrics such as precision, recall, accuracy, and F1-score. The classification algorithm is assessed based on four key terms: true positive (TP), true negative (TN), false negative (FN), and false positive (FP). These metrics are derived from these terms and serve as indicators to measure the effectiveness of pattern recognition. Figure 5 shows the chart of evaluation metrics.

Equation (2) illustrates accuracy (Acc), a performance criterion that shows how closely a calculation’s result matches the right value^60^.

2 $$\:\text{A}\text{c}\text{c}\text{u}\text{r}\text{a}\text{c}\text{y}\:=\frac{TP+TN}{TP+FP+TN+FN}.$$

Classifier accuracy includes other important factors, such as precision - the proportion of correctly detected targets among all detected targets. Furthermore, as Eqs. (3) and (4) show, recall - the number of successfully detected targets divided by the total number of known true targets - is present^61^.

3 $$\:\text{P}\text{r}\text{e}\text{c}\text{i}\text{s}\text{i}\text{o}\text{n}\:=\frac{TP}{TP+FP}$$

4 $$\:\text{R}\text{e}\text{c}\text{a}\text{l}\text{l}\:=\frac{TP}{TP+FN}.$$

The term F1-Score refers to the metric that evaluates the classification ability of an algorithm by taking into account both precision and recall. Equation (5) illustrates its definition, which is the harmonic mean of precision and recall^62^.

5 $$\:\text{F}1-\text{s}\text{c}\text{o}\text{r}\text{e}\:=\:\frac{2.\:\:Precision\:.\:\:Recall}{Precision+Recall}.$$

## Results and discussions

According to the used datasets for depression and Schizophrenia, therefore, their statistical significance can be illustrated in Table 3. In this study, the significance *P*-value has been chosen as (*P* < 0.05). Each dataset contains control cases (Normal) and condition cases (Patient). In case of depression, the patients have higher skewness value than the normal cases due to the heterogeneity in the motor activity pattern. While, it has much lower kurtosis that means the data is more flatter due to the abnormal activity pattern. The gender distribution has *P* = 0.158 which means the gender type does not have a significant effect on the results. In case of schizophrenia, the patients have higher skewness and kurtosis values than the normal cases while the gender distribution has *P* = 0.369. Since the *P*-value of all samples is less than < 0.05, the dataset distribution has a significant effect on the results.

**Table 3:** The datasets statistical features.

Illness	Condition			Control			*P*-value (All samples)
	Skewness	Kurtosis	*P*- value (Gender)	Skewness	Kurtosis	*P*- value (Gender)	
Depression	3.83	-2.67	0.158	0.76	-0.626	0.087	2.3e-4
Schizophrenia	4.22	3.11	0.396	1.1	0.87	0.074	1e-4

**Fig. 6:**
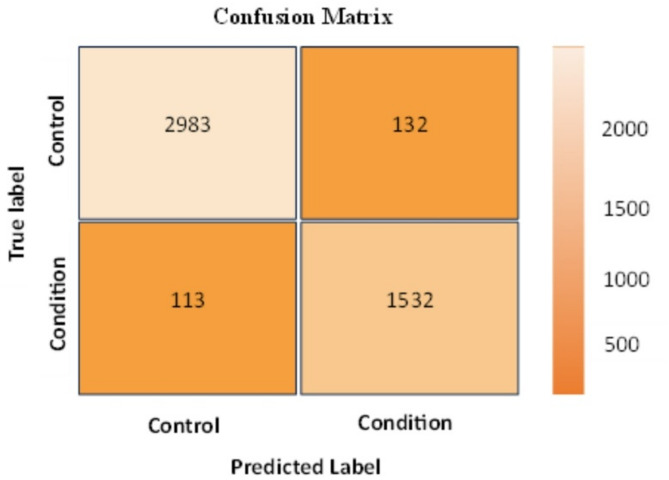
Confusion matrix of classification process for depression disorder.

**Fig. 7:**
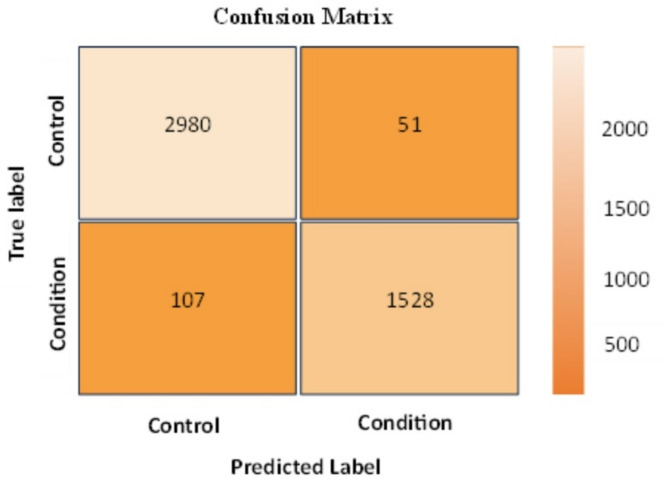
Confusion matrix of classification process for Schizophrenia disorder.

Every model has been trained with 80% of the data, and the remaining 20% has been used to test its performance. Table 4 lists the performance values for depression and schizophrenia disorders. Table 4 shows that the accuracy of the classification process for depression disorder is 94.85%, precision is 92.1%, Recall is 93.13%, and F1-score is 92.61%. In addition, the accuracy of the classification process for schizophrenia disorder is 96.6%, precision is 96.77%, Recall is 93.5%, and F1-score is 95.11%. A confusion matrix has been generated for each model to illustrate the classification results. Figures 6 and 7 present the confusion matrices for the classification of schizophrenia and depressive disorders, respectively.

**Table 4:** Performance values for illness.

Validation metrices	Depression	Schizophrenia
Accuracy	94.85%	96.6%
Precision	92.1%	96.77%
Recall	93.13%	93.5%
F1-Score	92.61%	95.11%

### Different encoding techniques

There are other encoding techniques that can be used for encoding images like Gramian Angular Field and Recurrence Plot. This section introduces different comparing with the MTF technique.

#### Gramian angular field

**Fig. 8:**
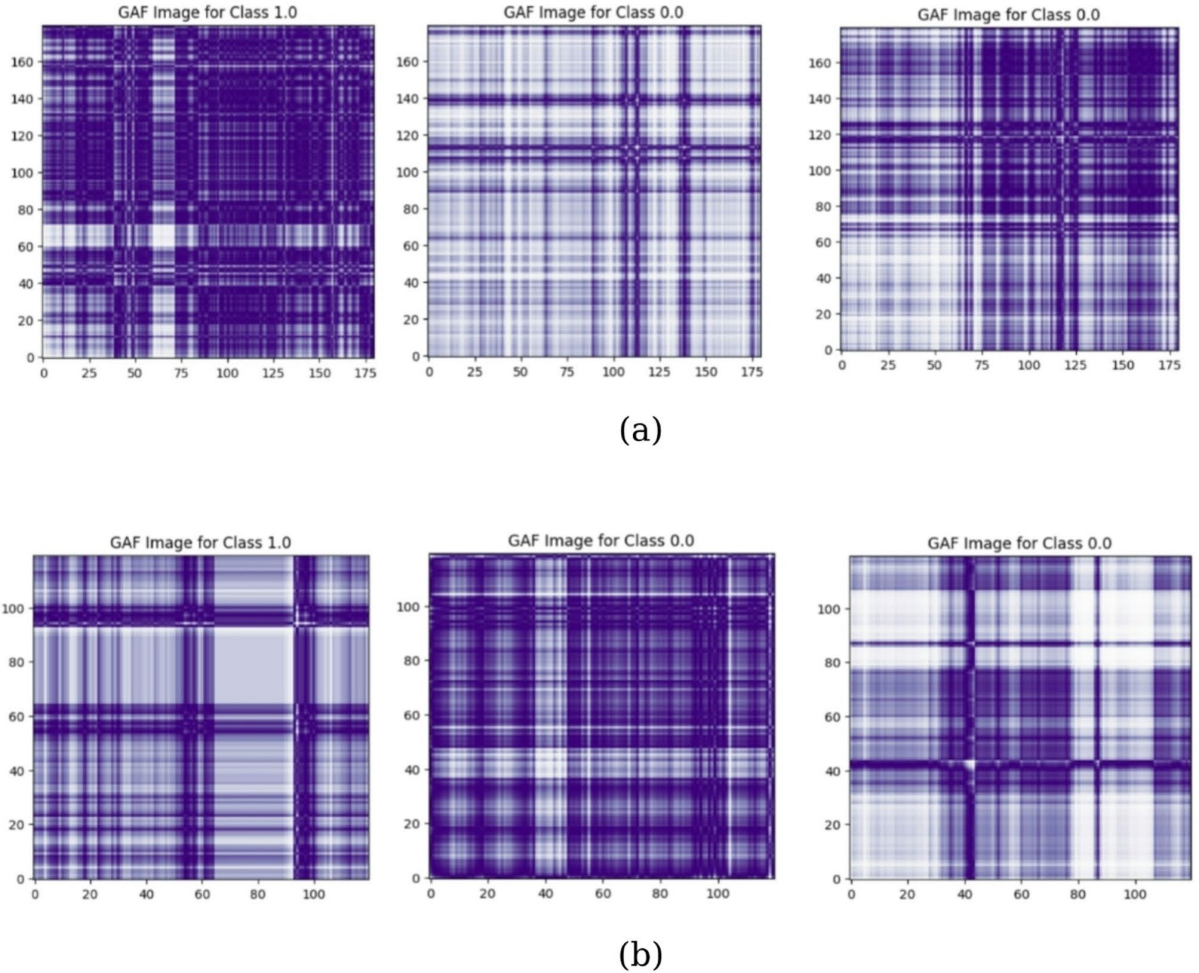
Samples of GAF images for (**a**) depression, (**b**) Schizophrenia.

The Gramian angular fields (GAF) convert each time series into images using a matrix based on polar coordinates. First, a Piecewise aggregate approximation is performed to reduce the length of time series for aligning them to same length then values are normalized between − 1 and 1. After normalization, these values ​​are transformed into polar coordinates, where the radial distance is specified by the time index, and the cosine of each value is specified by the angular component. The encoded data points move along concentric rings at different angular locations over time. A major advantage of this transformation, compared to Cartesian representations, is that it is bidirectional and preserves the entire temporal structure of the original sequence^52^. Samples of the GAF encoding technique for the depression and Schizophrenia datasets are shown in Fig. 8.

#### Recurrence plot

**Fig. 9:**
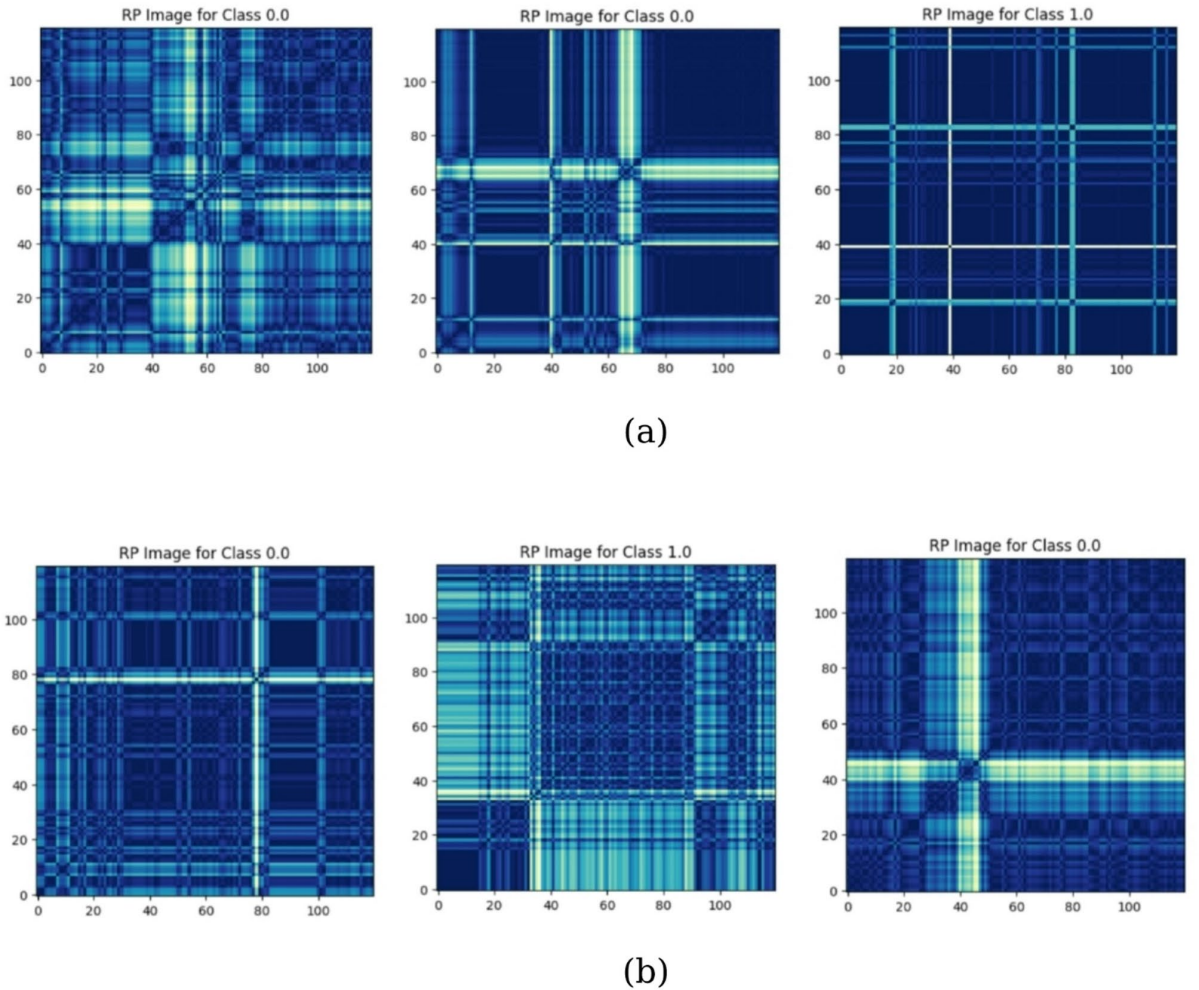
Samples of RP images for (**a**) depression, (**b**) Schizophrenia.

Recurrence Plot (RP) is an attractive method for encoding time series into visual patterns. This technique converts a one-dimensional time series into a two-dimensional image by calculating the similarity/distance between all pairs of points in the input time series. Simply put, it produces a square matrix whose elements indicate whether states at two points in time repeat to a predefined extent (or using a related distance function). The dark or light midtones of the final image can highlight periodic patterns, sharp transitions, or other hidden temporal details in the signal. Structured images, which represent time-related information, enable computer vision and deep learning paradigms (such as classifiers, object detection, or pattern recognition) to successfully apply time-related problems^63^. Samples of the RP encoding technique for the depression and Schizophrenia datasets are shown in Fig. 9.

Table 5 shows a comparison between the results of using MTF, GAF and RP for image encoding for depression and schizophrenia diseases. Table 6 shows the classification accuracy based on different models across distinct data segments (3 h, 1 h, 20 min) for depression and schizophrenia diseases, respectively.

Table 5 shows that the accuracy of the classification process for depression disorder using CNN to MTF encoding is 94.85%, precision is 92.1%, Recall is 93.13%, and F1-score is 92.61%. While the accuracy of the classification process using CNN to GAF encoding is 86.92%, precision is 81.18%, Recall is 85.19%, and F1-score is 83.13%. Also, the accuracy of the classification process using CNN to RP encoding is 89.55%, precision is 89.47%, Recall is 90.45%, and F1-score is 89.94%. In case of schizophrenia disorder, the accuracy of the classification using CNN to MTF encoding is 96.6%, precision is 96.77%, Recall is 93.5%, and F1-score is 95.11%. While the accuracy of the classification process using CNN to GAF encoding is 92.22%, precision is 91.41%, Recall is 92.6%, and F1-score is 92.04%. Also, the accuracy of the classification process using CNN to RP encoding is 94.5%, precision is 94.93%, Recall is 94.5%, and F1-score is 94.74%.

**Table 5:** Comparison between the MTF, GAF and RP results for image encoding.

Technique	Depression				Schizophrenia			
	Accuracy	Precision	Recall	F1-score	Accuracy	Precision	Recall	F1-score
Modified CNN to MTF images	94.85%	92.1%	93.13%	92.61%	96.6%	96.77%	93.5%	95.11%
Modified CNN to GAF images	86.92%	81.18%	85.19%	83.13%	92.22%	91.41%	92.6%	92.04%
Modified CNN to RP images	89.55%	89.47%	90.45%	89.94%	94.5%	94.93%	94.5%	94.74%

Table 6 shows that the accuracy of the classification process for depression disorder using 3 h segment is 85.47%, precision is 86.23%, Recall is 85.43%, and F1-score is 85.83%. While the accuracy of the classification process using 1 h segment is 80.35%, precision is 80.57%, Recall is 81.22%, and F1-score is 80.89%. Also, the accuracy of the classification process using 20 min segment is 74.62%, precision is 73.42%, Recall is 74.31%, and F1-score is 73.86%. In case of schizophrenia disorder, the accuracy of the classification using 3 h segment is 90.33%, precision is 90.53%, Recall is 91.34%, and F1-score is 91.28%. While the accuracy of the classification process using 1 h segment is 88.31%, precision is 89.16%, Recall is 88.31%, and F1-score is 88.73%. Also, the accuracy of the classification process using 20 min segment is 81.25%, precision is 82.14%, Recall is 81.25%, and F1-score is 81.69%.

**Table 6:** Classification accuracy based on different models across distinct data segments.

Segment	Depression				Schizophrenia			
	Accuracy	Precision	Recall	F1-score	Accuracy	Precision	Recall	F1-score
3 h	85.47%	86.23%	85.43%	85.83%	90.33%	90.53%	91.34%	91.28%
1 h	80.35%	80.57%	81.22%	80.89%	88.31%	89.16%	88.31%	88.73%
20 min	74.62%	73.42%	74.31%	73.86%	81.25%	82.14%	81.25%	81.69%

#### Comparative results

**Fig. 10:**
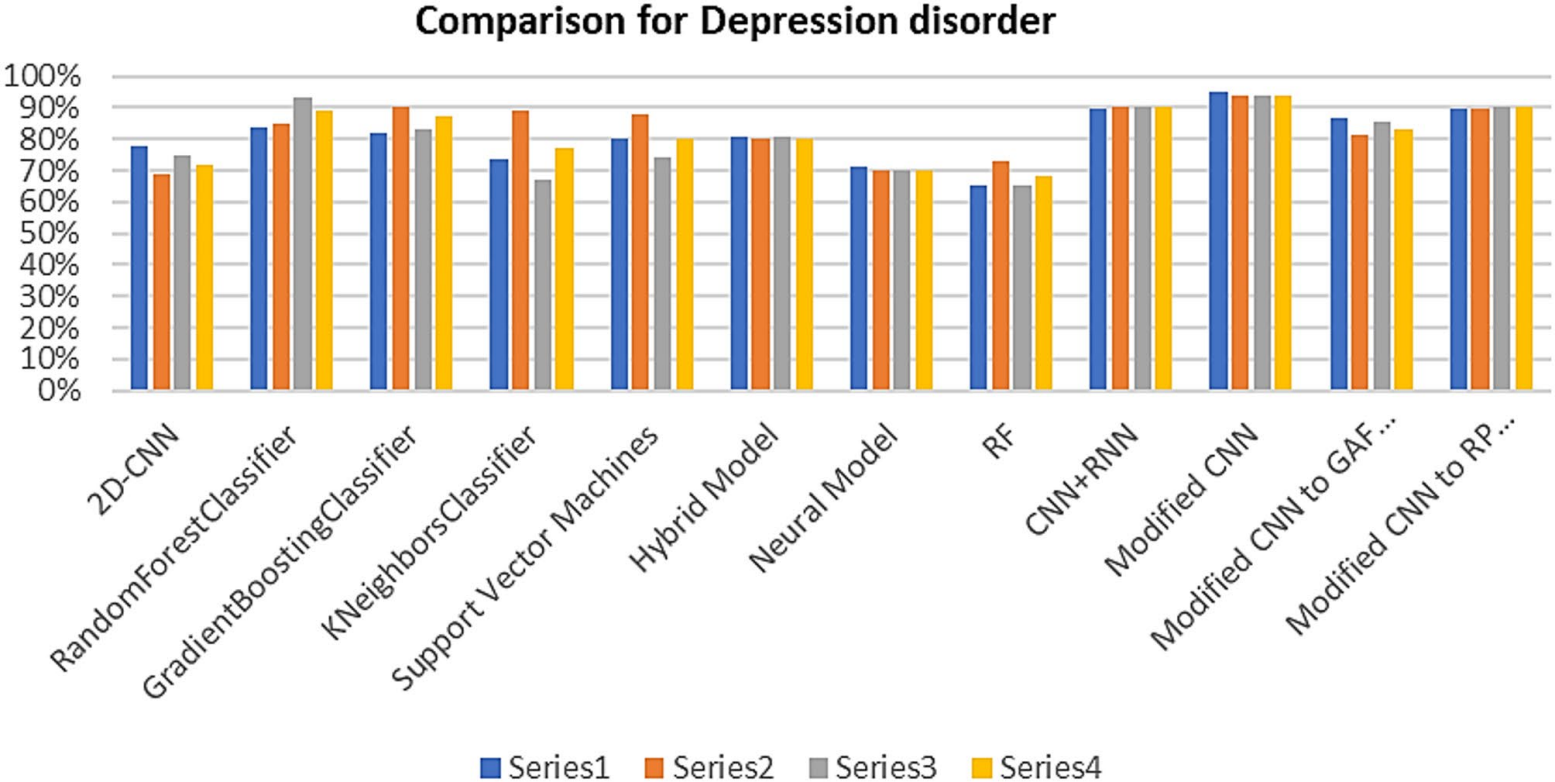
Performance comparison for depression disorder.

**Fig. 11:**
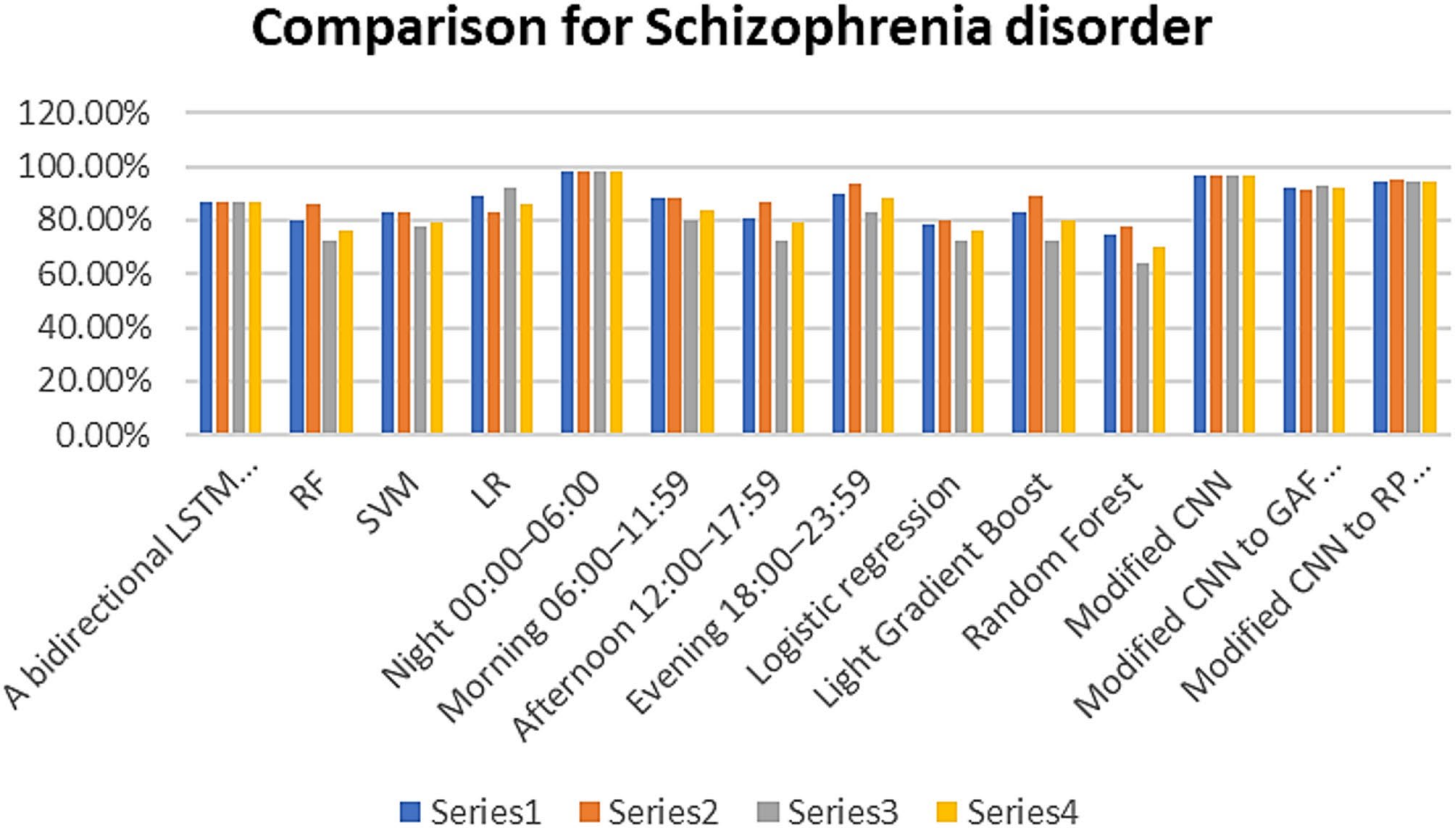
Performance comparison for Schizophrenia disorder.

To ensure the effectiveness of the proposed system, a comparative study between our works and other research using the same dataset (Depresjon and Psykose) is shown in Tables 7 and 8. The accuracy, precision, recall and F1-score are the main comparison topics. Figures 10 and 11 show the performance comparison for depression and schizophrenia disorders, respectively.

**Table 7:** Comparison between the proposed methodology and different research that used the same dataset for depression disorder.

Research	Technique	Accuracy	Precision	Recall	F1-score
^64^	ANN	90%	92%	86%	88%
^65^	2D-CNN	78%	69%	75%	72%
^29^	Random Forest	83.41%	85%	93%	89%
	Gradient Boosting	82.03%	90%	83%	87%
	K-Neighbors	73.27%	89%	67%	77%
	Support Vector Machines	80.18%	88%	74%	80%
^66^	Hybrid Model	81%	80%	81%	80%
	Neural Model	71%	70%	70%	70%
	RF	65%	73%	65%	68%
^67^	CNN + RNN	89.74%	90%	90%	90%
Proposed Method	Modified CNN to MTF images	94.85%	92.1%	93.13%	92.61%
Proposed Method	Modified CNN to GAF images	86.92%	81.18%	85.19%	83.13%
Proposed Method	Modified CNN to RP images	89.55%	89.47%	90.45%	89.94%

**Table 8:** Comparison between the proposed methodology and different research that used the same dataset for schizophrenia disorder.

Research	Technique	Accuracy	Precision	Recall	F1-score
^68^	A bidirectional LSTM network	86.60%	87.12%	87.03%	87.07%
^39^	RF	0.80 ± 0.09	0.86 ± 0.20	0.72 ± 0.11	0.76 ± 0.11
	SVM	0.83 ± 0.07	0.83 ± 0.14	0.78 ± 0.13	0.79 ± 0.08
	LR	0.89 ± 0.06	0.83 ± 0.15	0.92 ± 0.10	0.86 ± 0.08
^16^	Night 00:00–06:00	98.24%	98%	98%	98%
	Morning 06:00–11:59	87.97%	88%	80%	84%
	Afternoon 12:00–17:59	80.92%	87%	72%	79%
	Evening 18:00–23:59	89.84%	94%	83%	88%
^46^	Logistic regression	78.72%	80%	72.72%	76.18%
	Light Gradient Boost	82.97%	88.88%	72.72%	80%
	Random Forest	74.46%	77.77%	63.63%	70%
Proposed Method	Modified CNN to MTF images	96.6%	96.77%	93.5%	95.11%
Proposed Method	Modified CNN to GAF images	92.22%	91.41%	92.6%	92.04%
Proposed Method	Modified CNN to RP images	94.5%	94.93%	94.5%	94.74%

## Conclusion

Mental health issues remain a major public health, social and economic problem worldwide, and have always been identified using laborious and subjective technologies. wearable sensors have a great potential as a continuous mental health monitoring system. The development of wearable technology powered by AI is growing alongside the demand for mental health services. This study defined the actigraphy motor data obtained from patients with schizophrenia and depression using a feature engineering technique based on wrist-worn actigraphy data. The proposed used Depresjon dataset for depression disorder and Psykose for schizophrenia disorder. Because the two datasets are small, they are split into varied size of the time window (TW) and add all of them to augment the dataset to better capture activity changes. Markov Transition Field (MTF) is used to encode the time series dataset into images. Then, for the process of classification, two different models using Convolutional Neural Networks (CNN) with more Conv2D layers and Dropout layers to reduce overfitting are used. According to experimental results, this research outperformed the prior state-of-the-art in terms of classification accuracy, precision, and recall. The proposed system achieved an accuracy of 96.6% in schizophrenia and 94.85% in depression. Also, the precision, recall, and F1-score of the proposed system are 96.77%, 93.5%, 95.11% for schizophrenia and 92.1%, 93.13%, 92.61% for depression, respectively.

## Data Availability

The datasets generated and analysed during the current study are available in http://datasets.simula.no/depresjon/ and https://datasets.simula.no/psykose/ repository.
